# Rationing of Nursing Care and Patient Safety

**DOI:** 10.3389/fpsyg.2021.676970

**Published:** 2021-09-09

**Authors:** Izabela Witczak, Łukasz Rypicz, Piotr Karniej, Agnieszka Młynarska, Grzegorz Kubielas, Izabella Uchmanowicz

**Affiliations:** ^1^Department of Economics and Quality in Health Care, Faculty of Health Sciences, Wrocław Medical University, Wrocław, Poland; ^2^Department of Organisation and Management, Faculty of Health Sciences, Wrocław Medical University, Wrocław, Poland; ^3^Department of Electrocardiology, Upper Silesian Medical Centre, Katowice, Poland; ^4^Department of Gerontology and Geriatric Nursing, Medical University of Silesia, Katowice, Poland; ^5^Department of Clinical Nursing, Faculty of Health Sciences, Wrocław Medical University, Wrocław, Poland

**Keywords:** hospital care, unfinished nursing care, safety climate, patient safety, nursing

## Abstract

**Background:** Nursing care has a significant impact on patient safety, which affects clinical outcomes, patients’ satisfaction with the care received and nursing personnel’s satisfaction with the care provided. This study aimed to determine the extent of nursing care rationing and its relationship with patient safety including identification of the specific reasons.

**Methods:** This cross-sectional study involved 245 nurses and was performed between April–June 2019 in four hospitals in Wrocław, Poland. The standardized and relevant research tools such as Hospital Survey on Patient Safety Culture (HSOPSC) and the Perceived Implicit Rationing of Nursing Care (PIRNCA) were used. The data was submitted to hierarchical multiple regression analysis. The study was approved by the Bioethics Committee and was followed with the STROBE guidelines.

**Results:** The PIRNCA scores were negatively correlated with the HSOPSC subscales, which indicates that more frequent rationing of nursing care was associated with lower levels of patient safety parameters. It was shown that the highest level of unfinished nursing care was associated with decreases in patient safety factors linked with supervisor manager expectations actions promoting safety (rs = −0.321, *p* < 0.001), teamwork within hospital units (rs = −0.377, *p* < 0.001), feedback and communication about error (rs = −0.271, *p* < 0.001), teamwork across hospital units (rs = −0.221, *p* < 0.01), and hospital handoffs transitions (rs = −0.179, *p* < 0.01). Moreover, the strongest association was observed between the PIRNCA scores with patient safety grade (rs = 0.477, *p* < 0.001). Also, the PIRNCA scores among the internal unit were significantly higher than in the intensive care and surgical units.

**Conclusion:** Our study indicated the presence of nursing care rationing. Regarding patient safety, we found insufficient numbers of medical personnel and excessive personnel workload for providing safe care to patients, a lack of transparency in handling adverse event reports and analyses, and a lack of cooperation between hospital units regarding patient safety.

## Introduction

According to the Global Patient Safety Action Plan 2021–2030 by World Health Organization (WHO), patient safety is defined as *“a framework of organized activities that creates cultures, processes, procedures, behaviours, technologies and environments in health care that consistently and sustainably lower risks, reduce the occurrence of avoidable harm, make error less likely and reduce its impact when it does occur”* (World Health Organization, 2021).

Ensuring patient safety is an international challenge, as it affects every country in the world. Regardless of geography or economic standing, modern societies demand high quality, safety-oriented health care. Therefore, health center managers face the massive challenge of ensuring patient safety. The global challenge of ensuring health care safety and consolidating efforts associated with it is echoed in the current vision of the WHO, which refers to *“a world where every patient receives safe health care, without risks and harm, every time, everywhere”* (World Health Organization, 2017).

Contemporary theories of patient safety point to new types of risks associated with the near-constant pressure on health care organizations, staff shortages, excessive workload, large numbers of complex cases, the use of new technologies, and other factors. These stressors put middle management and first-line personnel in a tough position, at risk of breaching their standards and failing to provide optimum health care quality and safety (Thomas, 2020).

Nursing care has a significant impact on patient safety, which affects clinical outcomes, patients’ satisfaction with the care received and nursing personnel’s satisfaction with the care provided. It appears that nurses, faced with insufficient resources and urgent tasks, find it difficult or even impossible to meet all the requirements set out in individual nursing care plans. Even more alarmingly, nurses may shorten, delay, or even omit elements of the required patient care (Dhaini et al., 2019). A situation of staff shortage forces nurses to ration patient care and prioritize their interventions based on a clinical judgment. This may lead to their restricting or neglecting planned nursing care, which may increase the risk of negative patient outcomes (Rochefort et al., 2016). Consequently, the effects of nursing care rationing go against the principles of holistic nursing and adversely affects nursing care quality (Mandal et al., 2019).

Rationing of nursing care is a common problem in healthcare institutions and it can lead to many adverse events. Rationing of nursing care can be a contributor to reduced quality of patient’s care but also can lead to real threats to patient safety (Park et al., 2018). Missed nursing care is a consequence of many other aspects of the professional functioning of nursing staff. These include low job satisfaction, elevated stress levels, increased risk of burnout, higher work absenteeism, and increased staff rotation (Kalisch et al., 2014; Papastavrou et al., 2014b). Previous studies have identified several potential determinants influencing levels of rationing among nursing professionals (Młynarska et al., 2020). Moreover, it was found that rationing of nursing care also depends on psychological factors such as life satisfaction and life orientation. It is a fact that low levels of life satisfaction and a more pessimistic life orientation affect the higher occurrence of missed nursing care (Uchmanowicz et al., 2021).

The recent literature shows that nursing care rationing is a growing ethical concern in health care systems, as more and more commonly, nurses are forced to prioritize their activities and neglect the “lower priority” ones or leave them unfinished, which has adverse patient outcomes and decreased nurses’ job satisfaction (Scott et al., 2019). Ethical or moral dilemmas originated from care rationing prevent nursing staff to fulfill their tasks professionally according to ethical values. Jeopardizing the adoption of ethical actions in nursing care may arouse feelings of discomfort and distress as well as adverse consequences for both nurses and patients (Papastavrou et al., 2014a). Patient safety requires minimizing the risk of medication errors (a form of missed care) but also maximizing staffing quality. In the past, patient safety focused on medical errors, and did not fully account for shortcomings in nursing care, such as failing to provide full care timely, e.g., repositioning a patient position, medication dosing, and another care consideration (Dabney and Kalisch, 2015).

Nursing staff plays a crucial significant role in shaping patient safety culture. Safety culture is a multi-dimensional concept that comprises an assessment of leadership styles, individual and group ethical values, attitudes and behaviors, practicing evidence-based medicine, using communication channels, learning from mistakes, recognizing systemic shortcomings, and providing patient-centered care (Manzanera et al., 2018). So far, there is limited comprehensive evidence related to the implications of rationing nursing care on patient safety. There is a particular need for intra- and inter-institutional comparisons within health care institutions while also taking into account the measurement of rationing of nursing care, which contributes to the organizational culture of patient safety. Therefore, the aim of the study was to determine the extent of nursing care rationing and its relationship with patient safety.

## Materials and Methods

### Study Participants

The study was performed between April and June 2019 among nurses working in the four main hospitals in Wrocław (Poland), namely: University Clinical Hospital (UCH), Clinical Hospital of the Ministry of Internal Affairs and Administration (CHMIAA), Provincial Specialist Hospital (PSH), and Military Clinical Hospital with the Polyclinic (MCHP). The study included 245 participants, where 222 (90.6%) were nurses and 23 (9.4%) were midwifes. Two-hundred thirty-six (96.3%) of the participants were females and 9 (3.7%) were males. The work characteristics are presented in Table 1.

**TABLE 1:** Socio-demographic and work characteristics of nurses in the study (*N* = 245).

	Statistics	Frequency
Unit	Surgery department	65(26.5%)
	Internal	38(15.5%)
	ICU	51(20.8%)
	Others	91(37.1%)
Gender*	Female	236(96.3%)
	Male	9(3.7%)
Work hours per week	20–39 h	108(44.1%)
	40–59 h	101(41.2%)
	>60 h	36(14.7%)
Work experience	<5 year	81(33.1%)
	6–15 years	45(18.4%)
	16–20 years	38(15.5%)
	>21 years	81(33.1%)
Experience in the current work	<5 year	96(39.2%)
	6–15 years	53(21.6%)
	16–20 years	29(11.8%)
	>21 years	67(27.3%)
Staffing*	UCW	215(87.8%)
	CHMIAA	9(3.7%)
	PSH	8(3.3%)
	MCHP	10(4.1%)
Hospital size	<300 beds	0(0.0%)
	300–600 beds	9(3.7%)
	>600 beds	236(96.3%)

*The percentages do not sum up to 100, since the respondents did not answer the question.UCH, University Clinical Hospital; CHMIAA, Clinical Hospital of the Ministry of Internal Affairs and Administration; PSH, Provincial Specialist Hospital; MCHP, Military Clinical Hospital with the Polyclinic.

### Ethical Considerations

The STROBE (STrengthening the Reporting of OBservational studies in Epidemiology) guidelines for observational studies were followed. The study was carried out following the guidelines of the Declaration of Helsinki and Good Clinical Practice. Participation in the study was voluntary and anonymous. The study protocol was approved by the Bioethics Committee of Wrocław Medical University in Poland (permission no. KB–41/2019).

### Research Instruments

For the present study we used the following instruments: a demographic data sheet, the Hospital Survey on Patient Safety Culture (HSOPSC; Sorra and Nieva, 2013; Giai et al., 2017), and the Perceived Implicit Rationing of Nursing Care (PIRNCA) questionnaire (Jones, 2014).

### The HSOPSC Instrument

The HSOPSC instrument was used to assess a healthcare staff perspective on patient safety culture in hospital environments (Sorra and Nieva, 2013). We used the Polish version of HSOPSC (Sorra et al., 2016), which was adapted by Szpakowski et al. (2019). This is a questionnaire designed to assess patient safety culture at health care institutions. The Polish-language version of the HSOPSC is identical to the original version of the questionnaire and contains of 42 items. Each item is assessed from a hospital staff unit perspective by the 5-point Likert-type scale, which allows the individual to express how much they agree or disagree with a particular statement (from 1 = “strongly disagree” to 5 = “strongly agree”) or how often (from 1 = “never to” 5 = “always”) do the following things happen in their work area/unit. These 42 items are grouped into 12 composite measures: 10 safety culture dimensions and two outcome dimensions (question about an overall grade on patient safety for their work area/unit and the number of events they reported over the past 12 months).

Sorra et al. (2016) grouped 12 composites into 4 dimensions: (1) background variables; (2) outcome measures; (3) safety culture dimensions (unit level); and (4) safety culture dimensions (hospital-wide). The structure and reliability of the Polish version of HSOPSC was presented in Table 2. The validation study carried out by Szpakowski et al. (2019) among the nursing/midwifery staff (*N* = 103) indicated that the internal consistency of the 12 safety culture dimensions of Polish version of the HSOPSC was lower than the original US version (Duffy et al., 2018) and many European versions of the questionnaire (Smits et al., 2008; Hammer et al., 2011; Sarac et al., 2011; Robida, 2013; Perneger et al., 2014; Waterson et al., 2019). The reliability coefficients ranged from 0.38 for the subscale of “Organizational learning—continuous improvement and Communication Openness” to 0.89 for the subscale of “Frequency of events reported” (Szpakowski et al., 2019). The factors of safety culture with Cronbach’s alpha around or greater than 0.70 is considered as satisfactory. We included in our analysis the following dimensions of patient safety culture: (1) Frequency of events reported (three-items, α = 0.90); (2) Patient safety grade; (3) Supervisor/manager expectations and actions promoting patient safety (four items, α = 0.63); (4) Teamwork within units (4 items, α = 0.78); (5) Feedback and communication about errors (three items, α = 0.62); (6) Teamwork across units (four items, α = 0.72); (7) Handoffs and transitions (four items, α = 0.68) (Szpakowski et al., 2019).

**TABLE 2:** Descriptive statistics, structure and results of reliability analysis of 12 dimensions of the questionnaire (*N* = 245).

Dimensions	Number of items	Mean		Cronbach’s α reliability in this study (*N* = 245)	Cronbach’s α reliability Szpakowski et al. (2019) (*N* = 103)
		*M*	SD		
**Outcome measures**					
Frequency of events reported	3	10.58	3.39	0.90	0.89
Overall perceptions of patient safety	4	12.99	2.69	0.55	0.50
Patient safety grade	1	2.69	0.78	–	–
Number of events reported*	1	–	–	–	–
**Safety culture dimensions (unit level)**					
Supervisor/manager expectations and actions promoting patient safety	4	12.56	3.14	0.63	0.73
Organizational learning—continuous improvement	3	10.13	1.59	0.24	0.38
Teamwork within units	4	13.78	3.27	0.78	0.73
Communication openness	3	9.37	2.27	0.36	0.38
Feedback and communication about errors	3	10.05	2.59	0.62	0.84
Non-punitive response to errors	3	8.43	1.94	0.34	0.61
Staffing	4	10.70	1.91	−0.34	0.45
Management support for patient safety	3	9.27	1.93	0.57	0.68
**Safety culture dimensions (hospital-wide)**					
Teamwork across units	4	12.10	2.77	0.72	0.42
Handoffs and transitions	4	13.26	2.62	0.68	0.47

*Number of Events Reported was not reported because of the ordinal scales of measurement. The table does not consist Background Variables.

### The PIRNCA Questionnaire

Rationing of nursing care was assessed using the PIRNCA questionnaire (Jones, 2014). The PIRNCA inventory measures 31 nursing activities common to medical-surgical inpatient settings (Jones, 2014). Respondents were asked to rate the rationing frequency with which they were unable to complete within the previous seven working shifts on a four-point scale, where 0 = “never,”, 1 = “rarely,” 2 = “sometimes,” and 3 = “often,” The overall result of care rationing rate is the mean score taken over all the items. If none of these activities were required during these seven shifts, the respondent should response “not applicable.” The questions marked “not applicable” were excluded from the final result of PIRNCA scale. Thus, the total score ranges between 0 and 3, and may be interpreted as follows: higher scores indicate more perceived implicit rationing of nursing care. We employed the Polish version of PIRNCA adapted by Uchmanowicz et al. (2020) that confirmed the unidimensional structure and internal consistency of the Polish version of the measurement. The reliability of the Polish version of the tool assessed by Cronbach’s alpha indicating high reliability (α = 0.957) (Uchmanowicz et al., 2020). In this study, the Cronbach’s alpha yielded 0.977 and confirmed that the Polish version of PIRNCA is the reliable measure.

### Data Collection

This cross-sectional multicenter study used survey methods and convenience sampling method. The inclusion criteria were: work experience of over 6 months and consent to participate in the study. In turn, the exclusion criteria were: work experience under 6 months and lack of consent to participate in the study. All respondents received information about the study procedure and aims of the research and gave their informed consent to participate in the study with a guarantee of anonymity. The study used validated and standardized research questionnaires and an author-designed questionnaire to collect socio-demographic data. A total sample of 280 registered nurses who met the inclusion criteria to participate in the study was recruited for the study, of which a total group of 245 nurses was finally qualified. During the verification of the completeness of the collected questionnaires, a group of 35 nurses was excluded due to incomplete data. A total response rate was 87.5% which correspond with a missing data of 12.5%. Those nurses with missing data (excluded from analysis) were not significantly different from those who had full data including age, gender and work experience.

### Statistical Analysis

Qualitative data was presented in the form of numbers (n) and percentages (%). Quantitative data were presented in the form of mean (*M*) and standard deviation (SD). The normality of the distribution of continuous variables was assessed using the Shapiro–Wilk test. The non-normally distributed variables for the two groups were compared with the Mann–Whitney U-test and three or more groups were compared with the Kruskal–Wallis H test. The correlation between quantitative variables were analyzed using of the Pearson linear correlation coefficient (for normally distributed variables) or the Spearman’s correlation coefficient (for variables with distributions other than normal one). The following criteria for the evaluation of the correlation strength between the variables were used: ±1—Perfect; from ±0.9 to ±0.7—Strong; from ±0.6 to ±0.4—Moderate; from ±0.3 to ±0.1—Weak; and ±0—None (Dancey and Reidy, 2011).

A series of multiple regression analyses were performed using a hierarchical method to verify the possibility of predicting the variables linked with patient safety culture at healthcare institutions based on the degree of the perceived implicit rationing of nursing care measured by total PIRNCA scale. The regression analysis was performed by entering two separate blocks of independent variables. In the first block, the parameters linked with work characteristics were introduced to the regression analysis as a group of control variables: unit (Surgical, Internal, Intensive Care, and others), work hours per week (20–39 h, 40–59 h, >60 h), total work experience (<5 years, 6–15 years, 16–20 years, >20 years), experience in current work (<5 years, 6–15 years, 16–20 years, >20 years). All of these variables were measured on the ordinal scale and recoded using the Dummy Coding method (coded variables: 0 = no, 1 = yes). In the second block, all designed models included a predictor of nursing care rationing (the total PIRNCA score). This sequential order of entry was based on *a priori* hypothesis which stated that the additional variance of a particular patient safety component may be explained by the rationing of nursing care measured after accounting for the variance related to work conditions. Before running hierarchical regression, we examined multicollinearity between the predictors by estimating the variance inflation factors (VIF). If the VIF value are is greater than 4, there is a possible problem of multicollinearity (Hair et al., 2009). In our study, the VIF values ranged between 1.168 and 1.203 (see the value in Tables 3, 4). The level of significance was set at *p* < 0.05 in all statistical tests.

**TABLE 3:** The relationships between Unfinished care (PIRNCA score) and Patient Safety Culture (components of HSOPSC scale).

Components of the HSOPC scale	Unfinished care (PIRNCA score)
**I. Outcome measures**	
Frequency of Event Reporting	–0.043
Patient Safety Grade^a^	0.477***
Number of Events Reported	0.022
**II. Safety culture dimensions (unit level)**	
Supervisor manager expectations actions promoting safety	−0.321***
Teamwork Within Hospital Units	−0.377***
Feedback and Communication About Error	−0.271***
**III. Safety culture dimensions (hospital-wide)**	
Teamwork Across Hospital Units	−0.221**
Hospital Handoffs Transitions	−0.179**

In case of the lack of normality of distribution: non-parametric rho Spearman’s correlation coefficient. ^a^The higher score indicated worsening patient safety grade. **p < 0.01. ***p < 0.001.

**TABLE 4:** Summary of Hierarchical Regression Analysis for perceived implicit rationing of nursing care (PIRNCA) associated with outcome measures of Patient Safety Culture, Safety Culture Dimensions (Unit level), and Safety Culture Dimensions (Hospital-wide).

		B	β	t	95%CI	R	R^2^	ΔR^2^	R^2^_adj_	F	ΔF	VIF
**Step**	**DV: Frequency of events reported**											
1	Work characteristics					0.313	0.098		0.050	F(11,207) = 2.047*		
2	+PIRNCA	−0.029	−0.007	−0.092	−0.642–0.585	0.113	0.098	0.000	0.046	F(12,206) = 1.868*	ΔF(1,206) = 0.008	1.189
	**DV: Patient Safety Grade**											
1	Work characteristics					0.324	0.105		0.058	F(11,207) = 2.21*		
2	+PIRNCA	0.415	0.422	6.43	0.288-0.542	0.505	0.255	0.150	0.211	F(12,206) = 5.866***	ΔF(1,206) = 41.342***	1.177
**Step**	**DV: Supervisor/manager expectations & actions promoting safety**											
1	Work characteristics					0.524	0.274		0.236	F(11,208) = 7.150***		
2	+ PIRNCA	−1.108	−0.278	−4.507	−1.593 to −0.624	0.582	0.339	0.065	0.301	F(12,207) = 8.856***	ΔF(1,207) = 20.316***	1.195
	**DV: Teamwork Within Hospital Units**											
1	Work characteristics					0.348	0.121		0.075	F(11,208) = 2.604**		
2	+PIRNCA	−1.575	−0.377	−5.635	−2.126 to −1.024	0.488	0.238	0.117	0.194	F(12,207) = 5.386***	ΔF(1,207) = 31.751***	1.218
	**DV: Feedback and Communication About Error**											
1	Work characteristics					0.376	0.141		0.095	F(11,204) = 3.056**		
2	+ PIRNCA	−0.715	−0.217	−3.142	−1.163 to −0.266	0.426	0.181	0.040	0.133	F(13,208) = 3.746***	ΔF(1,203) = 9.875**	1.185
**Step**	**DV: Teamwork Across Hospital Units**											
1	Work characteristics					0.356	0.127		0.080	F(11,204) = 2.696**		
2	+ PIRNCA	−0.636	−0.178	−2.518	−1.134 to −0.138	0.392	0.153	0.026	0.103	F(12,203) = 3.064**	ΔF(1,203) = 6.339*	1.199
	**DV: Hospital Handoffs & Transitions**											
1	Work characteristics					0.274	0.075		0.026	F(11,206) = 1.525		
2	+ PIRNCA	−0.628	−0.187	−2.589	−1.105 to −0.150	0.323	0.105	0.029	0.052	F(12,205) = 1.996*	ΔF(1,205) = 6.703*	1.188

Number of Events Reported were not considered as dependent variables because of the ordinal scales of measurement. *p < 0.05. **p < 0.01. ***p < 0.001.

## Results

### Respondents’ Sociodemographic Characteristics

The study sample included 245 nurses (100%). Most were female (*n* = 236, 96.3%), and only 3.7% of respondents were male (*n* = 9). The largest group of respondents worked in the surgical unit (*n* = 65, 26.5%). Large numbers of respondents also worked in the ICU (*n* = 51, 20.8%), internal medicine unit (*n* = 38, 15.5%), and other units (*n* = 92, 37.1%). Most respondents worked 20–39 or 40–59 h per week (*n* = 108, 44.1%, and *n* = 101, 41.2%, respectively). Two largest groups had more than 21 years (*n* = 84, 33.2%) and less than 5 years of work experience overall (*n* = 81, 33.1%). The findings regarding experience in the current workplace were similar: the largest group had worked there between 1 and 5 years (*n* = 96, 39.2%), and the second largest, more than 21 years (*n* = 67, 27.3%). Detailed socio-demographic characteristics are shown in Table 1.

### Distribution of Answers of the Perceived Implicit Rationing of Nursing Care Questionnaire

The raw date including percentage distribution of answers obtained in PIRNCA by nurses in each question separately are presented in Table 5.

**TABLE 5:** Distribution of answers of the Perceived Implicit Rationing of Nursing Care (PIRNCA) questionnaire.

Question	Never (0)	Rarely (1)	Sometimes (2)	Often (3)	N/a or no answer	Mean
1	28.40%	22.22%	27.98%	0.00%	21.40%	0.99
2	31.28%	21.81%	28.40%	0.00%	18.52%	0.96
3	36.63%	20.58%	28.40%	0.00%	14.40%	0.9
4	34.57%	21.40%	25.51%	0.00%	18.52%	0.89
5	33.74%	24.69%	25.93%	0.00%	15.64%	0.91
6	39.09%	27.16%	15.23%	0.00%	18.52%	0.71
7	40.74%	15.64%	20.99%	0.00%	22.63%	0.74
8	35.39%	26.34%	21.81%	0.00%	16.46%	0.84
9	58.85%	17.28%	12.35%	0.00%	11.52%	0.47
10	40.33%	20.16%	12.35%	0.00%	27.16%	0.62
11	48.56%	20.16%	12.76%	0.00%	18.52%	0.56
12	47.33%	26.34%	13.58%	0.00%	12.76%	0.61
13	35.80%	20.16%	26.75%	0.00%	17.28%	0.89
14	44.44%	24.28%	20.99%	0.00%	10.29%	0.74
15	30.04%	21.40%	38.27%	0.00%	10.29%	1.09
16	37.86%	23.87%	28.40%	0.00%	9.88%	0.89
17	30.45%	25.93%	34.16%	0.00%	9.47%	1.04
18	47.33%	14.81%	23.46%	0.00%	14.40%	0.72
19	39.51%	19.75%	27.16%	0.00%	13.58%	0.86
20	34.98%	30.04%	21.81%	0.00%	13.17%	0.85
21	39.09%	26.34%	21.81%	0.00%	12.76%	0.8
22	20.99%	36.63%	28.40%	0.00%	13.99%	1.09
23	25.10%	26.34%	36.21%	0.00%	12.35%	1.13
24	27.16%	27.98%	22.22%	0.00%	22.63%	0.94
25	25.93%	33.74%	28.81%	0.00%	11.52%	1.03
26	33.33%	23.87%	31.69%	0.00%	11.11%	0.98
27	34.16%	25.51%	27.16%	0.00%	13.17%	0.92
28	44.03%	20.16%	23.05%	0.00%	12.76%	0.76
29	41.56%	20.99%	23.46%	0.00%	13.99%	0.79
30	38.68%	25.10%	24.69%	0.00%	11.52%	0.84
31	30.45%	30.45%	26.75%	0.00%	12.35%	0.96

### Analysis of Relationship Between Rationing of Nursing Care and Nurses’ Work Characteristics

There were statistically significant differences in unfinished care (PIRNCA score) in the relation to nursing units, *H*(3) = 22.132; *p* < 0.001. The results in the PIRNCA scores among Internal Unit (M_rank_ = 135.19) and “others” (M_rank_ = 131.76) were significantly higher than in Intensive Care Unit (ICU) (M_rank_ = 88.50) and Surgical Unit (M_rank_ = 95.24). Moreover, the nurses with experience in the current workplace from 16 to 20 years had the higher levels of unfinished care (M_rank_ = 141.95) than nurses working in the current workplace with the period of time equal or less than 5 years (M_rank_ = 100.56), *H*(3) = 8.837; *p* = 0.032 (see Table 6).

**TABLE 6:** Comparison tests (Kruskal–Wallis H Test or Mann–Whitney U test) on overall missed nursing care by nurses’ work characteristics.

			*M* (SD)	*M*_rank_	Statistics	*p* value	Pair wise comparison
Unfinished care (PIRNCA score)	Unit	(a) Surgery department	1.50 (0.64)	95.24	*H*(3) = 22.132	*p* < 0.001	(c–a) = 0.539
							(c–d) = −3.641***
		(b) Internal department	2.00 (0.76)	135.19			(c–b) = 3.227**
		(c) ICU	1.43 (0.72)	88.50			(a–d) = −3.316**
							(a–b) = −2.903**
		(d) Others	1.97 (0.89)	131.76			(d–b) = 0.260
Unfinished care (PIRNCA score)	Gender	Female	1.74 (0.80)	113.93	*U* = 772		
		Male	1.54 (0.95)	90.78		*p* = 0.296	
Unfinished care (PIRNCA score)	Work hours per week	(a) 20–39 h	1.70 (0.75)	111.46	*H*(2) = 0.564	*p* = 0.754	
		(b) 40–59 h	1.74 (0.88)	111.88			
		(c) >60 h	1.79 (0.79)	120.98			
Unfinished care (PIRNCA score)	Work experience	≤5 year	1.59 (0.73)	100.23	*H*(3) = 5.509	*p* = 0.138	
		6–15 years	1.92 (0.92)	125.71			
		16–20 years	1.89 (0.85)	124.69			
		>20 years	1.71 (0.77)	112.37			
Unfinished care (PIRNCA score)	Experience in the current work	(a) ≤5 year	1.56 (0.74)	100.56	*H*(3) = 8.837	*p* = 0.032	(a–d) = −1.209
		(b) 6–15 years	1.79 (0.92)	116.92			(a–b) = −1.419
							(a–c) = −2.918 **
		(c) 16–20 years	2.10 (0.82)	141.95			(d–b) = 0.256
							(d–c) = 1.897
		(d) >20 years	1.74 (0.74)	113.76			(b–c) = −1.635
Unfinished care (PIRNCA score)	Hospital size	<300 beds	–	–	*U* = 805	*p* = 0.727	
		300–600 beds	1.76 (0.57)	120.88			
		>600 beds	1.73 (0.82)	112.71			

**p < 0.01. ***p < 0.001. Not all patients had assessed all of the items.

### Analysis of Correlations Between Unfinished Care and Patient Safety

In the present study, the rationing of nursing care was negatively correlated with the most components of patient safety culture. The correlation results at the unit and hospital levels suggested that the higher extent of unfinished care reported by nurses were associated with decreases in patient safety factors linked with supervisor manager expectations actions promoting safety (*rs* = −0.321, *p* < 0.001), teamwork within hospital units (*rs* = −0.377, *p* < 0.001), feedback and communication about error (*rs* = −0.271, *p* < 0.001), teamwork across hospital units (*rs* = −0.221, *p* < 0.01) and hospital handoffs transitions (*rs* = −0.179, *p* < 0.01)— see Table 5. The strongest association was observed between the PIRNCA scores with patient safety grade (*rs* = 0.477, *p* < 0.001).

### Hierarchical Regression Analysis of Relationship Between Rationing of Nursing Care and Patient Safety Measures

Hierarchical regression was used to specify the factors measured by HSOPSC as a function of unfinished care (the total PIRNCA score) and work characteristics (the control variable). The results of regression are presented in Table 4.

#### Analysis of Frequency of Event Reporting

At stage one of the hierarchical multiple regression, it was found that the variables linked with work characteristics contributed significantly to the regression model, *F*(11,207) = 2.047; *p* = 0.026 and accounted for 9.8% of the variance of the Frequency of event reporting variable. Furthermore, the analysis indicated that inclusion of the Unfinished care variable had not explained an additional variation in Frequency of Event Reporting, ΔR^2^ = 0.000, Δ*F*(1,206) = 0.008; *p* > 0.05.

#### Patient Safety Grade

The analysis at stage one of multiple regression revealed that the parameters of work characteristics contributed significantly to the regression model, *F*(11, 207) = 2.21; *p* = 0.015, and accounted for 10.5% of the variance of the Patient Safety Grade. Inclusion of the Unfinished care variable into the model had explained an additional 15.0% of variance in the factor of Patient Safety Grade and made the significant change in R^2^ coefficient, ΔF(1, 206) = 41.342; *p* < 0.001. When all independent variables were included in the regression model, both parameters of work conditions and unfinished care were the significant predictors of the Patient safety grade variable; both independent variables accounted for 21.1% of the variance in predicting the factor of Patient Safety Grade. The β coefficient for the Unfinished care component was significant, β = 0.422, *t* = 6.43, 95%CI: (0.288; 0.542). This result indicated that there was a decrease of patient safety grade as the Unfinished care variable increased.

### Hierarchical Regression Analysis of Relationship Between Rationing of Nursing Care and Patient Safety at Unit Level

#### Supervisor/Manager Expectations and Actions Promoting Safety

The hierarchical multiple regression at stage one revealed that the variables linked with work characteristics contributed significantly to the regression model, *F*(11,208) = 7.150; *p* < 0.001, and accounted for 27.4% of the variation in predicting the factor of Supervisor/manager expectations and actions promoting safety (see Table 4). After including the Unfinished care variable there was an additional 6.5% of variation explained for the Supervisor/manager expectations and actions promoting safety variable; there was the significant change in the R^2^ parameter, ΔF(1,207) = 20.316; *p* < 0.001. The model was also significant after inclusion of all independent variables into the regression model, *F*(12,207) = 8.856; *p* < 0.001; the independent variables accounted for 30.1% of the variance in predicting the variable of Supervisor/manager expectations and actions promoting safety. The β coefficient for the Unfinished care was significant, β = −0.278, *t* = −4.507, 95%CI: = (−1.593; −0.624). This suggested that the variable of Supervisor/manager expectations and actions promoting safety decreased as the Unfinished care parameter increased.

#### Analysis of Teamwork Within Hospital Units

The hierarchical multiple regression at stage one revealed that the factors linked with work characteristics contributed significantly to the regression model, *F*(11, 208) = 2.604; *p* = 0.004, and accounted for 12.1% of the variation in the parameter of Teamwork within hospital units (see Table 4). The regression model with the Unfinished care variable explained an additional 11.7% of variation in Teamwork Within Hospital Units and made the significant change in the R^2^ parameter, Δ*F*(1,207) = 31.751; *p* < 0.001. The model with all independent variables included was significant, *F*(12,207) = 5.386; *p* < 0.001; the independent variables accounted for 19.4% of the variance in the factor of Teamwork Within Hospital Units. The β coefficients for the Unfinished care was significant, β = −0.377, *t* = −5.635, 95%CI: = (−2.12; −1.024). This regression analysis revealed that unfinished care was associated with patient safety, since the increase in Unfinished care decreased the levels of teamwork.

#### Analysis of Feedback and Communication About Error

The hierarchical multiple regression at stage one showed that the variable of work characteristics contributed significantly to the regression model, *F*(11,204) = 3.056; *p* = 0.001, and accounted for 14.1% of the variation in Feedback and communication about error (Table 4). By introducing the Unfinished care variable into the model there was an additional 4.0% increase of the variance explained in terms of Feedback and Communication About Error and the significant change in the R^2^ parameter, Δ*F*(1,203) = 9.875; *p* = 0.002. The model for all independent variables included was significant, *F*(13, 208) = 3.746; *p* < 0.001. The independent variables accounted for 13.3% of the variance in the factor of Feedback and Communication About Error. The β coefficient for the Unfinished care was significant, β = −0.217, *t* = −3.142, 95%CI: = (−0.163; −0.266) suggesting that the increased variable of Unfinished care decreases the Feedback and Communication About Error parameter.

### Hierarchical Regression Analysis of Relationship Between Rationing of Nursing Care and Patient Safety Culture at the Hospital Level

#### Analysis of Teamwork Across Hospital Units

The hierarchical multiple regression stage one showed that the variable of work characteristics contributed significantly to the regression model, *F*(11,204) = 2.696; *p* = 0.003, and accounted for 12.7% of the variation in Teamwork Across Hospital Units (see Table 4). The inclusion of the Unfinished care variable into the model explained an additional 2.6% of variation in Teamwork Across Hospital Units parameter and made the significant change in the R^2^ coefficient, Δ*F*(1,203) = 6.339; *p* = 0.013. The model for all independent variables included was significant, *F*(12,203) = 3.064; *p* = 0.001; the independent variables accounted for 10.3% of the variance in Teamwork Across Hospital Units. The β coefficient for the Unfinished care was significant, β = −0.178, *t* = −2.518, 95%CI: (−1.134 to −0.138). This suggested that the increase in the variable of Unfinished care decreases the levels of the Teamwork Across Hospital Units variable.

#### Analysis of Hospital Handoffs and Transitions

The last hierarchical multiple regression analysis at stage one revealed that the variable of work characteristics did not contribute to the regression model, *F*(11, 206) = 1.525; *p* = 0.124 (see Table 4). The inclusion of the Unfinished care variable explained an 2.6% of variation in predicting the factor of Hospital Handoffs and Transitions and changed the R^2^ coefficient significantly, Δ*F*(1,205) = 6.703; *p* = 0.010. The model was significant after including all independent variables, *F*(12, 205) = 1.996; *p* = 0.026; the independent variables accounted for 5.2% of the variance in Hospital Handoffs and Transitions. The β coefficient for the Unfinished care was significant, β = −0.187, *t* = −2.589, 95%CI: (−1.105; −0.029). The regression analysis showed that the increase in the variable of Unfinished care decreased the levels of the Hospital handoffs and transitions factors.

## Discussion

To the best of our knowledge, our study is one of the first that involves a population of Polish nurses and it also considered comprehensive statistical analyses including hierarchical regression analysis and providing evidence based on direct relationship between nursing care rationing and patient safety. The present study demonstrated that PIRNCA scores were negatively correlated with the HSOPSC subscales, which indicates that more frequent rationing of nursing care was associated with lower levels of patient safety parameters. It was shown that the highest level of unfinished nursing care was associated with decreases in patient safety factors linked with supervisor manager expectations actions promoting safety, teamwork within hospital units, feedback and communication about error, teamwork across hospital units, and hospital handoffs transitions. Moreover, the strongest association was observed between the PIRNCA scores with patient safety grade. Also, the PIRNCA scores among the internal unit were significantly higher than in the intensive care and surgical units.

Building patient safety in health care is crucial for improving health service quality and reducing the incidence of adverse events. Omissions due to nursing care rationing may be as significant for patient safety as medical errors (Griffiths et al., 2018). Nursing staff have a major impact on the quality of care, as they take part in most diagnostic and therapeutic processes. Thus, identifying nursing care rationing and factors associated with it is necessary to enable undertaking the necessary interventions with a view to optimizing nursing care and solving the problem of missed nursing care (Hernández-Cruz et al., 2017).

A cross-sectional study conducted in a 400-bed community hospital in the Mid-Atlantic Region indicated that the extent of missed nursing care was significantly greater within medica-surgical, telemetry and step-down units as compared to critical care units (Duffy et al., 2018). The present results of missed nursing care are consistent with other studies; for instance Duffy et al. (2018) indicated the higher risk of nursing care rationing observed in the conservative management unit as compared to critical and medical-surgical units.

Poor teamwork is often associated with an inadequate assignment of responsibilities, tasks, and functions to specific hospital units. In consequence, for instance, administrative personnel may fail to prioritize patient safety, believing that it is the sole responsibility of clinical staff (Listyowardojo et al., 2017). In the present study, we have shown that several dimensions of patient safety culture were negatively influenced by rationing of nursing care, namely: (i) “Patient Safety” that measures whether procedures and systems are in place at preventing errors and there are patient safety problems; (ii) “Teamwork within units” which examines whether nursing staff support each other and work together as a team. (iii) “Supervisor/manager expectations and actions promoting safety” which test whether supervisors/managers consider staff suggestions for actions toward patient safety; (iv) “Feedback and Communication About Error,” which tests whether staff is informed about errors that happen, given a feedback about changes implemented, and had discussions about ways to prevent errors, and (v) “Hospital Handoffs and Transitions” examines whether the important patient care information are transferred across hospital units and during shift changes (Duffy et al., 2018). These findings suggest that the factors of patient safety and rationing of nursing care are of particular importance as shown only by a few studies on this issue (Gurková et al., 2020; Vaismoradi et al., 2020).

Several reports have shown that organization and management of nurse work environment are linked with a problem of missed nursing care. A cross-sectional RN4CAST study across European hospitals showed that favorable work environments, lower patient to nurse ratios were associated with lower proportions of missed nursing care reported by nurses (Ausserhofer et al., 2014). Our results provide evidence that increased the risk of missed care rationing is associated with degraded nurse work environment along the dimensions of teamwork within hospital units and feedback and communication about error. A similar study by Ausserhofer et al. (2014) also indicated that poor teamwork was negatively correlated with implicit care rationing. The other study by Kalisch and Lee (2010) investigating the relationship between nursing teamwork and the missed care nursing care showed that the stronger teamwork resulted in less missed nursing care reported. Similarly, the previous reports (Ausserhofer et al., 2013) showed the negative relationship between unfinished nursing care measured by the Basel Extent of Rationing of Nursing Care inventory (Schubert et al., 2007) and patient safety climate measured by the Safety Organizing Scale (Vogus and Sutcliffe, 2007). A Lebanese study demonstrated that safety culture was the weakest in those areas where communication and staffing problems existed (Okuyama et al., 2018).

Zhu et al. (2019) in their mediation effect tested by structural equation modeling study involving 7,802 nurses and 5,430 patients found interrelationships between nurse staffing, rationing of nursing care and patient outcomes. Their findings suggest that nurse shortage plays a mediating role in the relationship between nurse staffing, missed nursing care, and patient outcomes, after controlling for hospital and patient factors. Rationing of nursing care appears to be both an increasingly recognized and relatively common phenomenon in nursing practice. Rationing can be applied to every decision that is made, which can affect patients’ different physical and psychological needs, as well as the quality of patient care and levels of patient safety. Care rationing is related to both the economic and ethical dimensions of nursing services, and an adequate identification of the causes and a proper understanding of the mechanisms of care rationing is crucial (Scott et al., 2019).

A systematic review by Mandal et al. (2019) demonstrated that rationing of nursing care is pervasive, embedded in the work environment and threatens the occupational health and philosophical foundations of nursing practice, and has serious implications for patient safety. Therefore, further research should consider the development of strategies aimed at reconsidering the organizational basis of the nursing profession from a holistic perspective. Moreover, considering the potentially serious consequences of rationing nursing care on patient safety and potential adverse events, it is important to discuss and establish a framework for safe, competent nursing care enabling nurses to provide an optimal level of service in order to improve patient safety (Tønnessen et al., 2020).

Improving safety requires an organizational culture that allows patients to participate in promoting safe care. Nurses, who are leaders on all levels of care, should have the required competences and resources to support and educate patients with regard to factors that affect care safety (Sahlström et al., 2019). To make this possible, management must be involved in promoting safety culture. This involvement should be reflected in motivating personnel to perform their work with no errors, rewarding this performance, and promoting best practices. Transparency between management, medical personnel, and patients with regard to care quality (including, among other factors, medical errors) is essential to developing a good patient safety culture (Gandhi et al., 2018). Hence the importance of a safety system, built upon tried and trusted procedures and guidelines contributing to the prevention of errors (dimension: “Overall perceptions of patient safety”).

Despite the fact that the study used a multicenter protocol and was conducted in four different hospitals, there is one major potential limitation of this study, which concerns the same academic center and the city of Wrocław, that enables the results obtained to be generalized to the whole country. Moreover, an objective assessment tools should be also considered in future research, which would require verifying the observed correlations in other hospitals in Poland.

## Conclusion

The present study evaluated the relationships between nursing care rationing and patient safety. Our findings suggest a clear relationship between both areas. This study shows that it is needed to raise staff awareness about patient safety and rationing of nursing care to identify strengths and areas for improvement patient safety culture. The present research also encourages to conduct comparisons within and across health care organization and measure rationing of nursing care which influences the patient safety culture within the organization.

Our findings have several implications for nurses’ work and patient safety policy. The identification of shortcomings regarding patient safety culture allows for undertaking the interventions with a view to its improvement. Reporting adverse events, performing root cause analyses, and learning from past mistakes to prevent their recurrence together form the foundation of safety systems in contemporary health care. Health care managers should focus on building a culture of trust and eliminating punitiveness in the workplace. Our study identified considerable deficits in communication across hospital units. Lack of teamwork and poor communication do not support a safety culture.

## Data Availability Statement

The original contributions presented in the study are included in the article/supplementary material, further inquiries can be directed to the corresponding author.

## Ethics Statement

The study was carried out following the guidelines of the Declaration of Helsinki and Good Clinical Practice. Participation in the study was voluntary and anonymous. The study protocol was approved by the Bioethics Committee of Wrocław Medical University in Poland (permission no. KB-41/2019).

## Author Contributions

IW contributed to acquisition of data, drafting of the manuscript, and critical revision of the manuscript. ŁR contributed to acquisition of data, analysis and interpretation of data, and drafting of the manuscript. PK and GK contributed to acquisition of data and drafting of the manuscript. AM and IU contributed to study conception and design, analysis and interpretation of data, drafting of the manuscript, and critical revision of the manuscript.

## Conflict of Interest

The authors declare that the research was conducted in the absence of any commercial or financial relationships that could be construed as a potential conflict of interest. The reviewer JR declared a shared affiliation, with several of the authors IW, ŁR, PK, GK, and IU to the handling editor at the time of the review.

## Publisher’s Note

All claims expressed in this article are solely those of the authors and do not necessarily represent those of their affiliated organizations, or those of the publisher, the editors and the reviewers. Any product that may be evaluated in this article, or claim that may be made by its manufacturer, is not guaranteed or endorsed by the publisher.
